# The organization and dynamics of viral factories

**DOI:** 10.1128/jvi.00892-26

**Published:** 2026-09-14

**Authors:** Kathryn Steele Brittain, Leonardo Martins Delfino, Otavio Henrique Thiemann, Juliana R. Cortines, Kristin N. Parent

**Affiliations:** 1 Department of Biochemistry and Molecular Biology, Michigan State University 123744, East Lansing, Michigan, USA; 2 Physics Institute of São Carlos, University of São Paulo, São Carlos, Brazil; 3 Department of Genetics and Evolution, Federal University of São Carlos 236041, São Carlos, Brazil; 4 Departamento de Virologia, Instituto de Microbiologia Paulo de Góes, Universidade Federal do Rio de Janeiro 341406, Rio de Janeiro, Brazil; Indiana University Bloomington, Bloomington, Indiana, USA

**Keywords:** viral factory, virus–host interactions, viral replication, *Nucleocytoviricota*

## Abstract

Viral factories (VFs) are dynamic, virus-induced microcompartments that serve as centralized hubs in the host cell for viral genome replication, transcription, and virion assembly. These structures employ unique viral mechanisms for remodeling cellular architecture to create specialized replication organelles and improve the efficiency of viral propagation. VFs exhibit striking structural and functional diversity among RNA and DNA viruses, from reoviruses and poxviruses to the *Nucleocytoviricota* phylum. Some are enclosed by host-derived membranes, while others exist as biomolecular condensates from liquid–liquid phase separation. VFs recruit host lipids, cytoskeletal elements, and metabolic enzymes, effectively reprogramming the intracellular environment to favor viral replication. This review provides a comprehensive examination of the molecular composition, ultrastructure, and biogenesis of viral factories across a wide range of viral lineages and host systems. We describe membrane-bound and phase-separated VFs and the mechanisms by which they hijack host machinery to create these replication organelles and explore viral strategies to shield replication intermediates from host immune responses. Additional emphasis is placed on the complex VFs formed by giant viruses in the *Nucleocytoviricota*, whose ability to spatially compartmentalize replication and transcription, exclude ribosomes, and recruit host mitochondria and membranes blurs the line between viral and cellular organization. By integrating findings from cell biology and evolutionary virology, this review proposes that viral factories offer a conceptual framework for understanding virus–host coevolution and provides new insights into how their organization may have shaped the emergence of eukaryotic complexity.

## INTRODUCTION

Viruses are “nonliving” microscopic infection machines with unique mechanisms for hijacking host cell pathways to produce viral progeny and often induce cell death. Viral factories (VFs) are a prime example of these mechanisms and are also referred to as replication compartments, viroplasms, or inclusion bodies. VFs are specialized structures observed in many viral systems that enhance replication efficiency and promote evasion of immune detection by creating an optimized microenvironment for stages of infection, such as genome replication, transcription, and virion assembly in the host (1, 2). VFs vary across classifications in factory size, distribution among the cell, protein composition, and properties of compartmentalization within the cell.

The presence among viruses of a central replication hub that is important in infection brings light to the potential evolutionary significance of VFs. DNA replication, genome packaging, and virion morphogenesis are often orchestrated within these structures in distinct spatial zones. The distribution of VFs is reminiscent of cellular compartmentalization, drawing intriguing parallels to eukaryotic organelles, like the nucleus (3–6). This evolutionary convergence is fundamental for our understanding of more elaborate and visually striking replication centers such as those of the *Nucleocytoviricota* phylum, also known as giant viruses (GVs). Many, if not most, GVs assemble expansive, membrane-bound factories that truly blur the boundaries between viral and cellular organization while also inspiring new questions about viruses and their complexity (1–3, 7–10). In fact, these viruses have large, complex genomes encoding elements of the translation system, such as DNA replication and repair enzymes, RNA capping machinery, and regulators of cytoskeletal dynamics (11). A striking example of this complexity is Tupanvirus, which encodes all 20 aminoacyl-tRNA synthetases (11, 12). Interestingly, GVs also carry genetic material coding for many hypothetical proteins of unknown function (13), highlighting our limited understanding of their infection mechanisms. We explore how viral compartmentalization reshapes virus–host interactions and the influence of viral factories on the emergence of eukaryotic cell complexity.

## STRUCTURE AND COMPOSITION OF VIRAL FACTORIES DURING INFECTION

**Fig 1:**
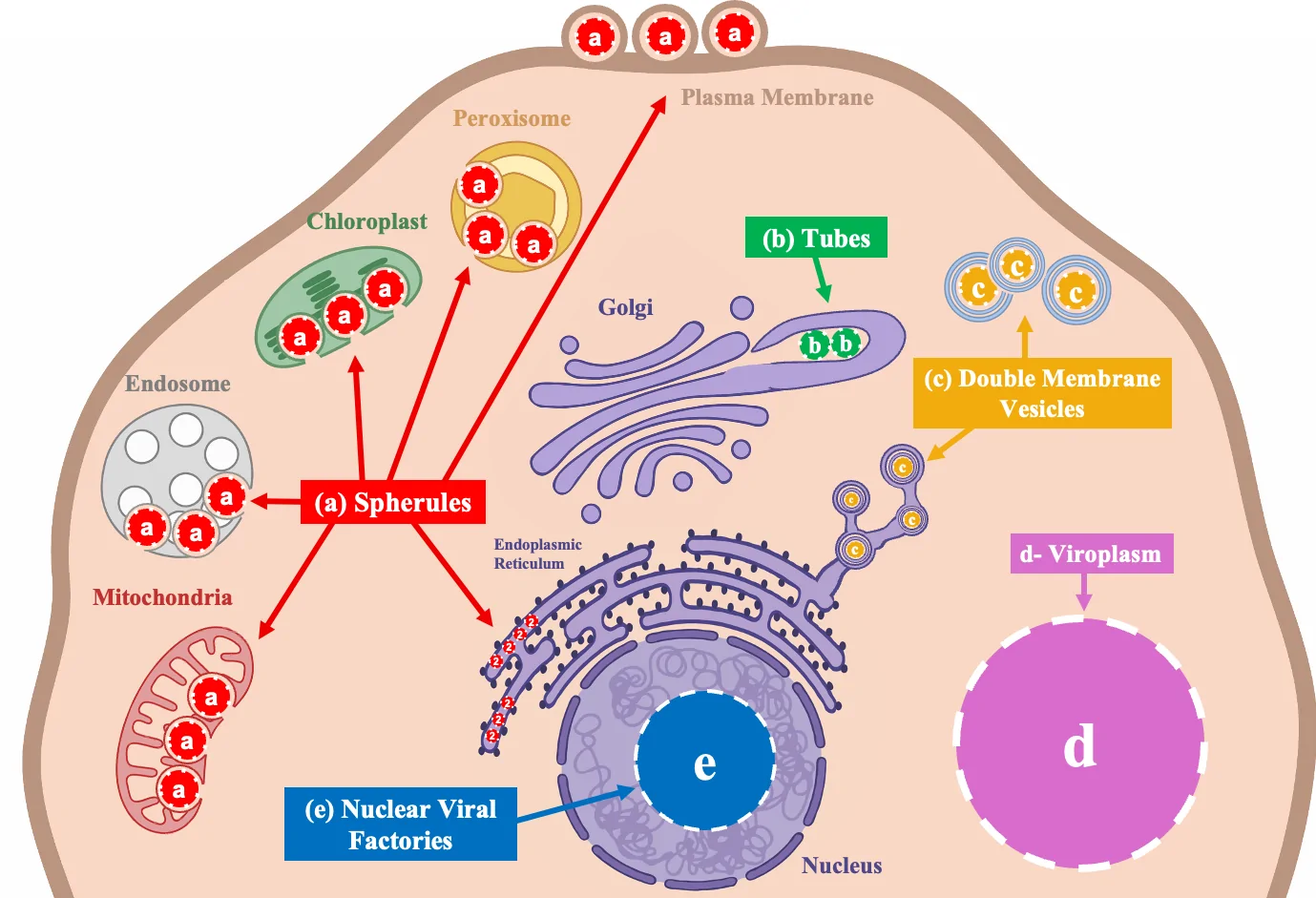
Different families of viruses hijack specific host organelles to establish the replication compartments observed in viral factories. These structures include (a) spherules, (b) tubes, (c) double-membrane vesicles, (d) viroplasms, and (e) nuclear viral factories, depending on the virus family and subcellular location. For example, tombusviruses and bromoviruses form spherules in the peroxisome and endoplasmic reticulum, respectively (17–23); alphaviruses form spherules at the plasma membrane (24–27); *Picornaviridae* and *Coronaviridae* generate double-membrane vesicles (28–31); *Bunyaviridae* produce tube-like structures attached to spherules (32–35); *Herpesviridae* and *Polyomaviridae* form nuclear viral factories (36–44); and *Poxviridae* and *Asfarviridae* develop cytoplasmic viroplasms (45–55). These specialized compartments support viral genome replication, transcription, and assembly, often altering host organelle architecture.

VFs are commonly composed of viral nucleic acids, replication enzymes, transcriptional machinery, structural proteins, and, in some cases, translation components or recruited host organelles (14–16). However, VFs exhibit unique structural features and compositional elements across viral classifications. Some include the presence of a defined membrane, variations in cellular localization, and differing utilization of host resources. Examples of these varied VF features are described next, and a summary of known VF organizations across viral families is provided in Table 1. The types of VFs are also depicted as a cartoon schematic in Fig. 1.

**TABLE 1:** Characteristics of eukaryotic VFs

Virus type	Type of viral factory	Cellular localization	Structure	Composition	References
Poxvirus (vaccinia)	Membrane-bound cytoplasmic factory, mini-nuclei, and viroplasms	Cytoplasm	Membrane (ER)	Viral DNA, ER membranes	(45, 49–51)
Flavivirus (dengue and Zika)	Vesicle packets, convoluted membranes	Endoplasmic reticulum (ER)	Membrane (ER)	Viral RNA, invaginated membranous structures	(56)
Coronavirus (SARS-CoV-2) *+*ssRNA	Double-membrane vesicles (DMVs)	ERGIC (ER-Golgi)	Membrane (ERGIC)	Viral RNA, DMVs, replication machinery	(28, 29, 57, 58)
Picornavirus (poliovirus)	Remodeled replication organelles	Golgi and ER	Membrane (PI4P enriched)	Viral RNA, membranes enriched with PI4P	(59)
Reovirus	Viroplasms	Cytoplasm	LLPS	Viral RNA, λ3 polymerase, μ2, σ2 proteins, microtubules, ER membranes	(60, 61)
Rabies virus −ssRNA	Negri bodies	Cytoplasm	LLPS (membraneless)	Nucleoprotein (N), phosphoprotein (P), genomic RNA	(62, 63)
Measles virus	Dynamic liquid inclusions	Cytoplasm	LLPS (membraneless)	N–P interaction, dynamic fusion/fission	(64)
Mimivirus	Giant viroplasms	Cytoplasm	Complex/mixed	Viral DNA, transcription enzymes, structural proteins, host factors	(5, 65)
Asfarvirus (African swine fever virus)	Perinuclear viral factory	Perinuclear (cytoplasm)	Likely membrane bound	Viral DNA, mitochondria, ribosomes, viral proteins	(46–48, 52–55)
Bunyavirus	Tube-like structures attached to spherules	Golgi	Membrane	Viral RNA, viral replication machinery, viral proteins	(32–35)
Tombusvirus	Spherules	Peroxisomes and ER	Membrane (ER)	Viral RNA, viral replication machinery, viral proteins, mitochondrial membranes	(17–19)
Bromovirus	Spherules	ER	Membrane (ER)	Viral RNA, viral replication machinery, viral proteins	(20, 21, 66)
Alphavirus (Chikunguya virus)	Spherules	Plasma membrane	Membrane (ER)	Viral RNA, viral replication machinery, viral proteins	(24–27)

## RNA VIRAL FACTORIES

**Fig 2:**
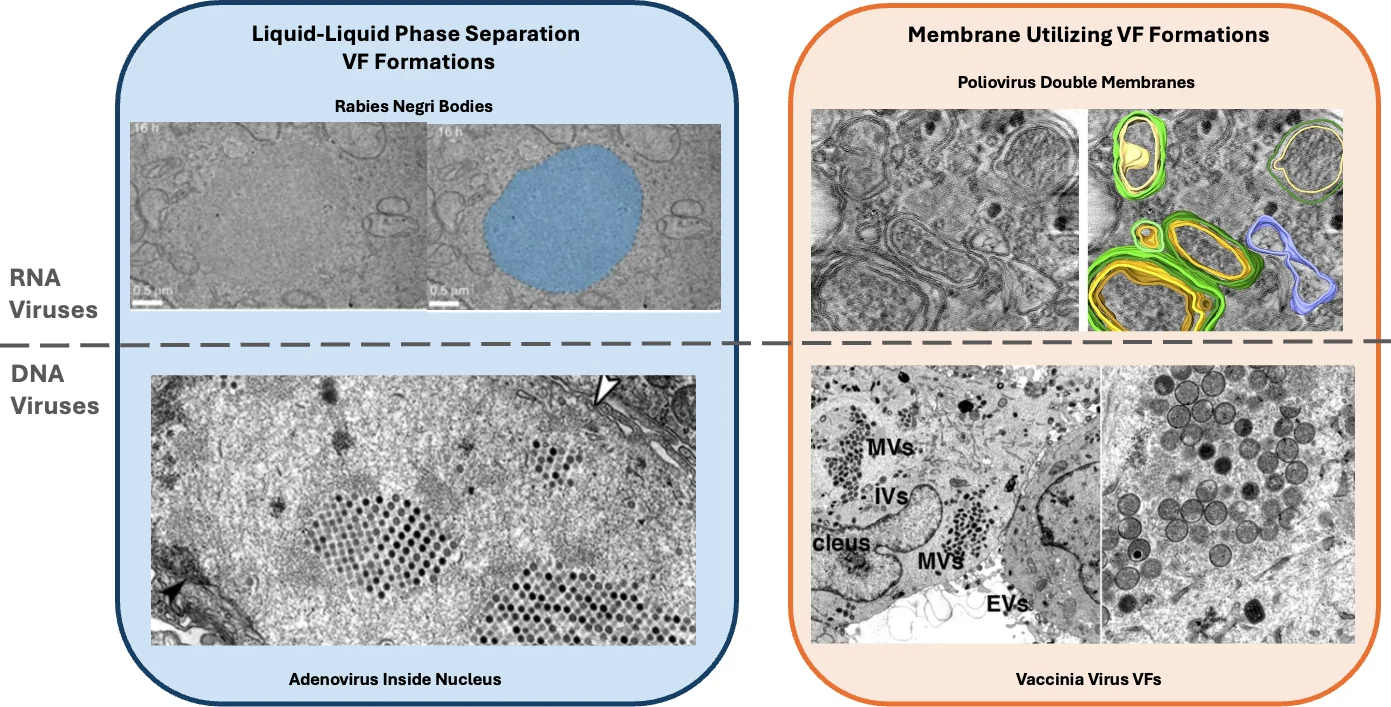
Examples of viral factories in diverse systems seen from electron microscopy imaging. Here we choose to highlight some diverse viral factory formations that utilize different mechanisms of organization. In the blue bubble (left side of the figure), we see examples of VF formation from liquid–liquid phase separation (LLPS). LLPS VF is observed from RNA viruses, including cytoplasmic Negri body formation in rabies-infected cells (top), which display electron-dense granular structures. The panel on the left is the original image, and the panel on the right highlights the Negri body with a blue circle. In addition, DNA viruses such as adenovirus demonstrate paracrystalline virus arrays forming inside the nucleus (bottom). In the orange bubble (right side of the figure), VFs can also be organized by enclosed membranous structures, and this can occur in both RNA and DNA viruses. For example, in RNA viruses such as poliovirus, infected cells were visualized by electron tomography, where the image in gray tones shows the original raw micrograph, and the colored areas show 3D volume segmentation that highlights membranes specifically. Both single-membrane structures (blue segmentation) and double-layered membrane structures (yellow/green) are seen in cells during the late stages of virus infection. In DNA poxviruses such as vaccinia virus, colocalization of VFs and continuous connections between endoplasmic reticulum membranes and viruses are observed. The top left panel is modified from Nikolic et al., *Nature Communications* (62), previously published under a Creative Commons Attribution 4.0 International License. The bottom left panel is modified from Jerman et al., *Viruses* (81), previously published under a Creative Commons Attribution 3.0 Unported License. The top right panel is modified from Belov et al., *Journal of Virology* (82). The bottom right panel is modified with permission from Weisberg et al., *Proceedings of the National Academy of Sciences* (51).

Almost all known RNA viruses assemble in the cytoplasm (57). VFs in RNA viruses are often composed of host-derived membranes or biomolecular condensates formed by liquid–liquid phase separation (LLPS) (Fig. 2) (14, 16). For example, host-derived lipid membranes are observed in many RNA viral infections, such as flaviviruses, which are +ssRNA viruses. Infections with both flaviviruses, dengue and Zika virus, induce endoplasmic reticulum (ER) membrane invaginations. These invaginations are noticeably in the rough ER, forming both vesicle packets and convoluted membranes to serve as replication hubs (56, 67). Poliovirus, a member of the *Picornaviridae* family, also forms replication complexes at the rough ER (59). Rearrangement of the rough ER likely provides access to ribosomes for viral RNA to undergo subsequent translation (56). Additionally, picornaviruses and some flaviviruses (e.g., HCV) restructure the Golgi and ER membranes to form replication organelles enriched in phosphatidylinositol-4-phosphate, a direct binding partner of the viral RNA-dependent RNA polymerase, RdRp3D^pol^ (68). It is thought that this interaction drives the RNA-dependent RNA polymerase RdRp3D^pol^ to the replication organelle and promotes stability for further viral replication complex activity (68). Additionally, cytosolic lipid droplets and membrane vesicles are also important drivers of viral replication in hepatitis C virus (HCV, *Flaviviridae* family) infection, specifically forming a “membranous web” that is likely related to the formation of viral replication factories (69). Flock house virus (dsRNA) replication induces similar changes but in the intramembrane space of the mitochondria (70–72). Furthermore, all known coronaviruses, also +ssRNA (e.g., SARS-CoV, SARS-CoV-2, MERS-CoV), generate double-membrane vesicles (DMVs) derived from the ER-Golgi intermediate compartment (ERGIC), where viral RNA synthesis takes place (28, 29, 57, 58). *Bunyaviridae* (−ssRNA), like Bunyamwera virus, employs Golgi membranes that form interactions with the mitochondria as viral replication compartments (32). These compartments consist of a tubular structure attached to a spherule (33). Interestingly, alphaviruses, like Chikungunya virus and Semiliki virus of the *Togaviridae* family (+ssRNA), form membranous replication organelles called spherules at the plasma membrane (24, 73, 74). Spherule replication compartments are also observed in *Tombusviridae* (+ssRNA) and *Bromoviridae* (+ssRNA) infection, where they localize to the peroxisomes and ER of the host cell, respectively (17–23, 66).

Some RNA viral components also undergo LLPS, forming biomolecular condensates and concentrating viral proteins and RNA without membrane encapsulation (Fig. 2). In addition to DMVs in SARS-CoV-2 infection, its nucleocapsid protein undergoes LLPS, facilitating RNA packaging in a distinct second factory site within the cell (75). *In vitro* experiments using SARS-CoV-2 nucleocapsid and phosphoproteins demonstrate that such condensates facilitate rapid capsid assembly, a process involving intrinsically disordered viral proteins interacting with the genome (75). A similar mechanism is observed in the −ssRNA viruses, vesicular stomatitis virus and Ebola virus (EBOV), where VF formation is also driven by LLPS (76–78). Ebola viral factories are largely composed of the EBOV nucleoprotein (77). Measles virus (−ssRNA) induces liquid-like inclusions through interactions between the nucleoprotein and phosphoprotein, exhibiting dynamic fusion and fission (64). Additionally, rabies virus (−ssRNA) forms Negri bodies, which are cytoplasmic aggregates of viral nucleoprotein, phosphoprotein, and genomic RNA (62, 63). *Reoviridae* are dsRNA viruses that also appear to form viral inclusions via LLPS, as observed in rotaviruses (79). Interestingly, electron microscopy revealed membranous materials within reovirus VFs, suggesting a scaffolding role in the replication cycle (60). Reoviruses assemble VFs with LLPS properties; however, their membranes come from remodeled ER (61). *Birniviridae* are also dsRNA viruses that form viral inclusions via LLPS (80). Through the biogenesis of specialized viral factories, RNA viruses achieve a critical isolation of their replicative core. This strategic partitioning not only evades host immunosurveillance but also centralizes the assembly apparatus, thereby ensuring the efficiency and success of the infectious cycle.

## DNA VIRAL FACTORIES

VFs are also observed in DNA virus infections, having nuclear and/or cytoplasmic morphologies (57). There are several examples of both dsDNA and ssDNA viruses that form VFs in the nucleus (Fig. 2). For example, herpes simplex virus 1 (dsDNA) forms nuclear factories, specifically localizing at the ND10 structures of the host cell nucleus (36, 37). On the other hand, Epstein–Barr virus (dsDNA) forms nuclear replication sites via LLPS, as the viral transcription factor EBNA2 and its coactivator EBNALP have intrinsically disordered regions that seemingly drive this separation (83). Another example of a DNA virus that can be cited includes H-1 parvovirus, an ssDNA virus that forms replication compartments in the nucleus (84). Similar mechanisms are observed in dsDNA adenoviruses (85–89). Papillomaviruses (dsDNA) are also thought to form replication compartments in the nucleus, as their genome is shown to associate with promyelocytic leukemia nuclear bodies upon entering the nucleus (90, 91), and the small dsDNA Polyomaviruses form tubular structures in the nucleus that seemingly serve as the site for viral replication (38).

VFs are also observed in the cytoplasm during infection (Fig. 2). In fact, cytoplasmic VFs are hallmarks of many nucleocytoplasmic large DNA virus (NCLDV) infection mechanisms, a category of viruses encompassing many large DNA viruses, including GVs. For example, a member of the *Poxviridae* family, the dsDNA vaccinia virus, has its DNA replication occurring in the distinct sites of the cytoplasm, specifically within membranes derived from the ER (92–94). Interestingly, these membranes coincide with successful DNA replication and seemingly dissipate in later-stage VFs when shifting into virion assembly. Furthermore, these VFs likely form via LLPS due in part to the H5 protein, a phenomenon observed in other H5 homologs of the *Poxviridae* family (45). An additional proposed model for replication compartmentalization is suggested for *Mirusviricota* with genomic data hinting at a nuclear-to-cytoplasmic conversion of viral replication (95). Cytoplasmic viral factories of giant viruses are further described in detail later in this review.

## ADDITIONAL CONTRIBUTIONS TO VF FORMATION

In addition to utilizing host organelles for membrane production and LLPS in the cytoplasm, host pathways and cellular components further influence VF formation. For example, host-derived lipids in picornavirus factories and cholesterol in HCV (dsDNA) contribute to envelope formation (30, 96). Additionally, host cells provide replication complexes not encoded by the viral genome, such as ribosomes (96). Host cytoskeletal elements can be critical for VF formation in some viruses. For example, the intermediate filament vimentin is important for structural formation and integrity of VFs in African swine fever virus (ASFV, dsDNA) and also Ebola virus (−ssRNA) (97, 98). A diverse array of host factors involved in transcription and replication is recruited to the site of viral replication to support infection (99). To sustain viral genome replication, capsid protein production, and virion assembly, VFs impose significant metabolic stress on the host cell, triggering profound metabolic reprogramming, including upregulation in purine and pyrimidine biosynthesis and an increase in glycolysis and ATP generation (100, 101). In fact, the energy needed for these stages of infection may explain the recruitment of mitochondria to the periphery of VFs as seen in ASFV (102). Furthermore, host lysosomal pathways are also utilized in VFs. Specifically, in reovirus infection, modified lysosomes can be used in later stages of VFs to transport mature virions to the plasma membrane for subsequent release (103). While some of these shifts serve specific viral needs, others are byproducts of disrupted cellular homeostasis. The full spectrum and regulation of these changes remain incompletely understood.

Viruses can also hijack known host intracellular degradation pathways to further promote viral infection mechanisms of VFs. Specifically, some viruses may utilize host autophagic proteins, as observed in the dsDNA human cytomegalovirus (HCMV) of the *Herpesviridae* family (104). Specifically, it was observed that host human foreskin fibroblast cell autophagic proteins L3 and SQSTM1 were localized with viral proteins of HCMV cytoplasmic viral assembly compartments, which are observed in later stages of infection (104). Host aggresomal pathways are also thought to influence VF formation due to similarities observed between aggresomes and VFs. In addition to the vimentin filaments observed in both aggresomes and VFs, ASFV VFs assemble in close proximity to the nucleus and microtubule organizing center and recruit mitochondria to the VF periphery (46–48, 97). Lastly, while GVs utilize host cell factors to accomplish viral factory formation, they present with an impressive genetic repertoire that seemingly equips them to manipulate host cells in favor of viral infection (105, 106). In fact, genes specifically involved in responses to oxidative stress, cell apoptosis pathways, and DNA repair were identified across both *Mimiviridae* and *Phycodnaviridae* viruses (107).

## COMPARTMENTALIZATION OF VIRAL GENOME OBSERVED IN PHAGE INFECTION

While VFs are associated with the infection of eukaryotic cells, it is interesting to highlight unique mechanisms of compartmentalization in bacteriophage infection of prokaryotic hosts. Specifically, giant bacteriophages, also called jumbo phages, such as 201φ2-1 and ΦKZ, of the *Chimalliviridae* family, infect *Pseudomonas* spp. and form a protein shell that physically separates the viral genome from host replication and transcription machinery and host restriction endonucleases (108, 109). These viral structures are made of chimallin A, which assembles as a single-layer lattice around the replicating viral genome, resembling a mesh-like “protein shell,” or phage nuclear enclosure (110). In the case of 201φ2-1, the structure is highly organized by gp105, which self-dimerizes and forms head-to-tail tetramers that reorganize as a stable mesh with pores of ~2 nm (111, 112). This shell serves as a selective barrier in that it physically excludes host ribosomes, thereby spatially separating viral transcription from translation, and also blocks access by host CRISPR-Cas nucleases, protecting the viral genome from immune attack (109).

In tandem with this form of compartmentalization, the jumbo phage infection process orchestrates spatial reorganization of the host cytoplasm. Notably, tubulin-like PhuZ filaments assemble in the host cytosol, serving the dual functions of positioning viral replication machinery mid-cell and guiding newly synthesized empty capsids to the surface of the protein shell for viral genome packaging (113). This PhuZ-mediated trafficking system ensures orderly assembly and spatial distribution of progeny virions (113). Interestingly, in *Chimalliviridae* phage infection, it was recently discovered that lipid membranes surround phage DNA while the phage nucleus forms, drawing parallels to eukaryotic viral infection (114). These structures and organization tactics in phage infection, while not considered true VFs, are a tribute to the importance of compartmentalization in viral infection.

## THE *NUCLEOCYTOVIRICOTA* VIRAL FACTORIES

**Fig 3:**
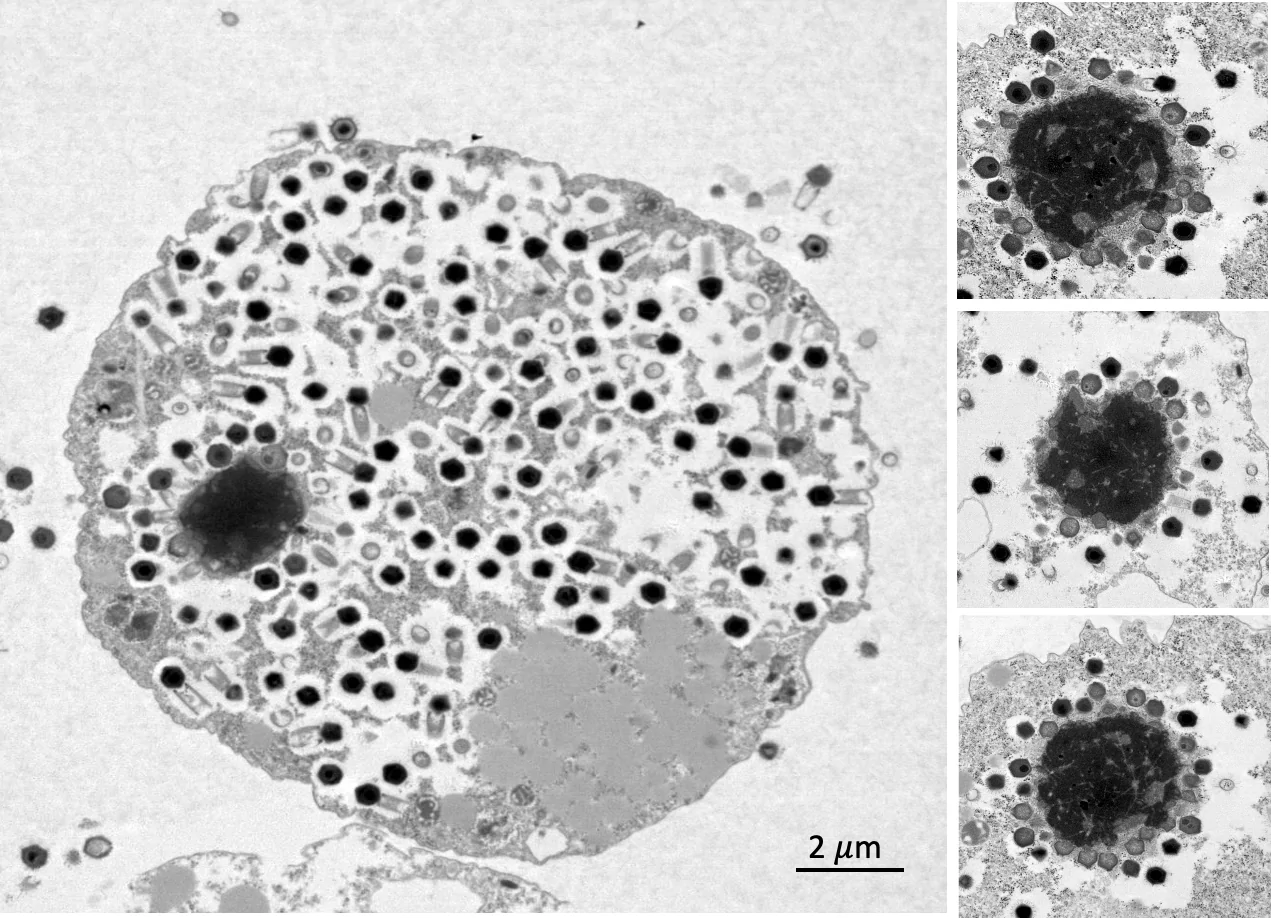
Transmission electron microscopy image of Tupanvirus of the *Mimiviridae* family of giant amoeba-infecting viruses (scale bar = 2 µm). The left panel is an entire cell, and the right panel shows three zoomed-in views of the VFs. Image credit: Kathryn Steele Brittain and Alicia Withrow.

While the structure and origin of VFs vary across classifications, they share core principles in the formation of organized replication centers for downstream infection. This convergence sets the stage for understanding the more elaborate and visually striking replication centers formed by GVs, which establish expansive, membrane-bound factories within the host cytoplasm (3) (Fig. 3). These VFs challenge classical boundaries between viral and cellular organization (115, 116). In the next section, we delve into how these extraordinary systems, hallmarks of the NCLDVs, reveal new paradigms in virology and provide unique insights into the evolution of complex cellular structures. These structures not only coordinate genome replication and virion morphogenesis but also shed light on evolutionary strategies used by viruses to adapt to and manipulate eukaryotic host metabolism. Despite major progress in ultrastructural and functional characterization, many aspects of GV VF composition and dynamics remain poorly understood. In the following sections, we provide a comprehensive overview of viral factories across *Nucleocytoviricota* families, highlighting conserved features, specific adaptations, and outstanding questions relevant to viral pathogenesis and evolution.

The discovery and subsequent investigations of the *Nucleocytoviricota* phylum have significantly expanded our understanding of virus-induced cell remodeling and replication. A defining feature of these viruses is their ability to form prominent cytoplasmic VFs, which serve as dedicated hubs for viral transcription, genome replication, and particle assembly (3). Although many VFs across the *Nucleocytoviricota* share features such as mitochondrial recruitment and spatial exclusion of the host nucleus, their organization and functional characteristics remain distinct and virus specific. The emergence of these VFs is thought to overcome the spatial constraints of intracellular trafficking, which becomes inefficient for large genomes (>300 kbp) and complex virus assembly (3, 117). While GVs may differ in the specifics of VF organization and especially complexity, they still share interesting features with their smaller counterparts. For example, recently, it has been suggested that mimivirus VFs are also formed via LLPS (118).

In the following sections, we explore how individual giant viruses implement these shared principles through unique but converging structural solutions. We provide examples of some of the most well-studied giant virus VFs, but this listing may not reflect every giant virus as new members of the NCDLV are being discovered rapidly.

## MIMIVIRIDAE

Mimivirus, a member of the *Mimiviridae* family of GVs, was first identified in 1992 as a gram-positive bacterium in samples from a water tower in Bradford, England (119). In 2003, it was reclassified as a virus after 16S rRNA sequencing failed and imaging revealed a 400 nm virion that seemingly infected amoeba (119). Since then, other GVs of the *Mimiviridae* family have been isolated, including Samba virus and, more recently, Tupanvirus from niche environments in Brazil (12, 120). Both experimental isolation from the environment and culture-independent metagenomic surveys reveal the worldwide prevalence of the *Mimiviridae* family (121–123).

VFs are a characteristic and critical feature of the *Mimiviridae* infection pathway, being detected as early as 4 hours post-infection (h.p.i.) (5). Among the giant viruses, this family’s viral organelle is the most well characterized and supports all stages of viral morphogenesis (5, 65). Acanthamoeba polyphaga mimivirus-isolated VFs indicated the stargate vertex is the first to be assembled and released from within the VF. Even though it is not directly within the viral factory, protein translation occurs in its vicinity (4, 118, 124), which suggests a supporting role during these crucial events. In Fig. 3, the elaborate VF of the *Mimiviridae* family member, the tailed-giant Tupanvirus, is shown with budding virions in various stages of maturation.

## MARSEILLEVIRIDAE

The *Marseilleviridae* family was first described in 2009 and includes viruses with relatively compact genomes (~370 kbp) enclosed within 250 nm icosahedral capsids, one of the smallest members of the giant viruses (125). Following phagocytosis and cytoplasmic delivery, a diffuse VF emerges at ~4 h.p.i., initiating transcription and replication. Virion assembly then progresses over the subsequent 8–12 hours. The VF can occupy up to half of the host cytoplasm and is often associated with vesicles harboring mature virions (126, 127).

A hallmark of Marseillevirus replication is the use of vesicle-mediated transport and packaging. Up to 1,000 progeny virions are enclosed within mono- or bilayered vesicles, forming large bundles destined for release approximately 24 h.p.i. (128). The Marseillevirus genome is organized with virus-encoded histones, which localize to the VF and may serve as scaffolds for viral genome condensation (129). In Melbournevirus and Noumeavirus, components of the host nuclear transcription machinery are transiently recruited from the nucleus to the VF.

In Jyvaskylavirus, a related family member, DNA is packaged via an incomplete fivefold vertex within the VF, while the host nucleus remains intact throughout infection (130). Virion assembly frequently takes place at the VF periphery, with internal vesicles likely aiding in particle transport for release. Interestingly, endosome-derived membranes are observed in VFs and seemingly contribute to the inner membranes observed in subsequent particles, further indicating complex VF–host interplay (126, 127).

## PANDORAVIRIDAE

Pandoraviruses were first isolated in 2013 and immediately drew attention due to their unprecedented particle size, genome complexity, and morphology that more closely resembles a bacterial cell than a traditional virus. The amphora-like virions reach up to 1 µm in length and enclose linear double-stranded DNA genomes ranging from 1.9 to 2.5 Mbp. Remarkably, ~70% of their predicted protein-coding genes are ORFans, highlighting their enigmatic evolutionary origin (121, 131).

Unlike most members of the *Nucleocytoviricota*, Pandoraviruses lack a canonical major capsid protein, and virion morphogenesis occurs within a translucent cytoplasmic viral factory occupying ~30% of the infected cell cytoplasm (132). Mitochondria and membrane structures of diverse origins, including lysosome-like organelles, are recruited into the VF interior, making Pandoravirus VFs structurally distinct among NCLDVs.

During Pandoravirus infection, the host nucleus and nucleolus progressively disintegrate as the VF matures. Assembly of new virions occurs within the VF, virions are observed as early as 6 h.p.i., even in the absence of host cell lysis, suggesting the use of exocytic pathways for viral release (133). Although structural and proteomic studies of the Pandoravirus VF remain limited, these features underscore its distinctive adaptation among giant viruses.

## PITHOVIRUS, CEDRATVIRUS, AND ORPHEOVIRUS

*Pithovirus sibericum*, discovered in 2014 in 30,000-year-old Siberian permafrost, sparked significant interest due to its unusual morphology and size. The virions are approximately 1.5 µm long and 0.9 µm wide and possess cork-like plugs that are released during infection to release the genome. Like pandoraviruses, pithoviruses lack a canonical major capsid protein, and their genome is packaged within a dense inner compartment (134, 135).

Cedratviruses and orpheoviruses are closely related but exhibit distinct structural features. Cedratviruses possess two corks at opposite poles of the virion, whereas orpheoviruses lack corks altogether but display surface fibrils and denser internal material.

During infection, cedratviruses and orpheoviruses form large cytoplasmic viral factories that appear electron-lucent and are located near the host nucleus. Interestingly, the host nucleus remains intact, while organelles such as mitochondria and lysosomes are actively recruited toward or within the VF. In cedratvirus infections, mitochondria accumulate both inside and around the VF (136).

Virion morphogenesis initiates centrally and progresses toward the VF periphery, possibly assisted by host membrane systems. As with Pandoraviruses, exocytosis-like release of virions has been proposed based on vesicle accumulation and the absence of classical lytic release. Despite their sparse genomic annotations, these viruses contribute to our understanding of VF diversity and evolution among giant viruses (136).

## MOLLIVIRUS

Molliviruses were discovered in the same 2014 Siberian permafrost sample as *Pithovirus sibericum* (137). Their spherical virions measure approximately 600 nm in diameter and are surrounded by two to four concentric layers of fibrils. The replication cycle begins when *Acanthamoeba* spp. phagocytose vesicles containing multiple virions. This is followed by vesicle fusion and the cytoplasmic release of viral cores. Within a few hours post-infection, Mollivirus forms a large, translucent viral factory near a deformed but present host nucleus. Virions at various stages of assembly are observed within the VF by 8 h.p.i (138). Mitochondria and other host organelles are displaced to the cell periphery, and the mitochondria themselves often appear elongated, potentially reflecting a stress-induced morphological adaptation (139).

Inside the Mollivirus VF, numerous vesicles of unknown origin, possibly derived from the Golgi or ER, accumulate and aid in capsid morphogenesis (138, 140). Unlike other NCLDVs, Mollivirus does not exhibit strong spatial separation of assembly zones within the VF. DNA is packaged before full capsid closure, and assembled virions are distributed throughout the factory.

The periphery of the VF contains a dense, fibrillar material of unknown origin that may be involved in late-stage assembly. Proteomic and structural data on the VF remain limited, but evidence suggests a replication strategy that combines features of currently observed NCLDV factories and unique mechanisms not previously observed (137, 138).

## YARAVIRUSES

Yaraviruses represent one of the most enigmatic possible members of the *Nucleocytoviricota* phylum. Described in 2020, these viruses have small, icosahedral virions (~80 nm) and a genome with over 90% ORFan genes, an unprecedented proportion among giant viruses (141, 142). Despite their small size and reduced genome, Yaraviruses retain core features associated with VF-based replication.

After entry, Yaraviruses induce a translucent cytoplasmic VF where virion morphogenesis occurs. The assembly process begins centrally, with crescent-shaped capsid precursors gradually maturing as they migrate toward the VF periphery. Both genome packaging and maturation seem to follow this spatial progression within the viral organelle (142).

Host organelles, including mitochondria, are found near or within the VF. Although data are limited, the replication cycle of Yaraviruses suggests a streamlined, yet conserved, NCLDV replication strategy, possibly reflecting evolutionary reduction from a more complex ancestor (142).

## EXTENDED ASFARVIRUSES: FAUSTOVIRUS, PACMANVIRUS, AND KAUMOEBAVIRUS

Extended members of the *Asfarviridae* clade, including Faustovirus, Pacmanvirus, and Kaumoebavirus, share a phylogenetic relationship with ASFV, the sole classified member of *Asfarviridae* (143). Despite their genomic diversity and partial structural divergence, these viruses follow a replication strategy consistent with other *Nucleocytoviricota* members, prominently featuring VFs.

Upon infection, large, electron-lucent VFs form adjacent to the host nucleus. These factories, which can exceed 3 µm in size, support both virion assembly and genome replication. Host mitochondria are often recruited to or remodeled near the VF periphery. Empty capsids and assembly intermediates, frequently observed as crescents, are distributed within the VF matrix (144).

Fully assembled particles appear geometrically organized around the VF, although precise mechanisms of capsid maturation and genome packaging remain unclear. Observations from electron microscopy indicate that these viruses use conserved principles for capsid assembly and DNA encapsidation, while adapting their structural output to diverse hosts (145).

## THREATS TO VIRAL FACTORIES

Host cells have developed defense systems against the viral factories of RNA and DNA viruses, and even compartmentalization mechanisms observed from some phage infections. For example, mouse cytomegalovirus, a dsDNA herpesvirus, is not efficient at infecting human cell lines, as humans are not its observed host (146). The limiting factor for this infection system seemingly stems from the E2F3 transcription factor and subsequent expression of FAM111B, a protease that also accumulates in VFs and is likely influencing VF formation and function (146). Furthermore, it is also shown that Simian Virus 40 variants (SV40), a dsDNA polyomavirus, with mutations in the large T-antigen and adenovirus infections, are restricted by the host cell nuclear protein FAM111A, which also localizes with SV40 VFs (147, 148). FAM111A and FAM111B are suggested to influence VF formation via proteolysis, given their proteolytic properties (147, 148). VFs may also be promising targets for drug discovery and development. For example, in ASFV (dsDNA), which infects pigs and directly impacts the pig industries in countries of Eastern Africa and Asia, analogs of deoxythymidine competitively inhibit viral DNA replication and subsequent VF formation (149).

Interestingly, the large cytoplasmic factories of GVs are susceptible to small dsDNA viruses, called virophages, that utilize their VFs for replication (150). The first reported virophage, Sputnik, was discovered along with mamavirus, a dsDNA virus in the *Mimiviridae* family, from a water tower in Paris (150). Sputnik utilizes the large cytoplasmic VFs in mamavirus infection to replicate, ultimately leading to fewer infectious mamavirus progeny likely due to changes in individual particle morphology, like thickening capsids and capsids containing only Sputnik progeny (150).

Finally, while compartmentalization of viral components promotes infection, it is also observed in host cell defense mechanisms against phage infection of *Bacillus subtilis* (151). Specifically, the YjbH protein in *B. subtilis* can bind to and accumulate the phage genome at a unique site in the cell while also interacting with the host FtsE protein to trigger cell division pathways (151). This leads to either the production of only one infected daughter cell per round of division or simply phage DNA surrounded by the host cell membrane (151).

## *NUCLEOCYTOVIRICOTA* VIRAL FACTORIES: THE EUKARYOGENESIS HYPOTHESIS

The structural and functional sophistication of *Nucleocytoviricota* VFs has also led to provocative theories on the origin of the eukaryotic nucleus. One of the “viral eukaryogenesis” hypotheses proposes that an ancestral NCLDV-like virus infected an Asgard archaeon, establishing a long-term symbiotic relationship that eventually gave rise to the nucleus (152). First articulated by Philip Bell in 2001, this idea has since been further developed (153, 154), where viruses contributed key innovations such as the decoupling of transcription and translation, mRNA capping with 7-methylguanosine (m7G), and possibly early components of the nuclear pore complex. These viral traits would have conferred selective advantages by protecting transcripts from host degradation and facilitating more regulated gene expression. Recent genomic and structural studies of giant viruses have strengthened this theory. Mimiviruses and related *Nucleocytoviricota* encode components typically associated with eukaryotic information-processing systems, including RNA polymerases, helicases, mRNA capping enzymes, translation-related genes such as tRNAs and aminoacyl-tRNA synthetases (12, 155), and even a translation initiation complex homologous to eukaryotic eIF4F (156). These viruses form cytoplasmic viral factories that are spatially and functionally compartmentalized, separating transcription from translation, mimicking a primitive nucleus (6). The existence of m7G caps in Mimivirus transcripts and the exclusion of ribosomes from the viral factory interior reinforce this analogy. Some models propose a multi-phase process in which the VF evolved into a nucleus, followed by the symbiotic acquisition of mitochondria and development of the endomembrane system (152). Alternative models suggest multiple origins: for instance, Medusavirus replicates inside the host nucleus and encodes eukaryotic-like histones, raising the possibility that nuclear evolution involved more than one viral lineage or strategy (157, 158). Whether the eukaryotic nucleus arose from a viral structure (as a viral factory fossil) or through viral gene domestication remains debated. Nonetheless, the viral eukaryogenesis hypothesis offers a compelling framework for how viruses may have shaped the origin of cellular complexity. It also opens the door to rethinking the evolutionary boundaries between viruses and life.

## CONCLUSION

In summary, VFs represent a remarkable evolutionary innovation enabling diverse viruses, from small RNA viruses to giant DNA viruses, to orchestrate complex intracellular replication processes. While they vary widely in organization, composition, and localization, viral factories consistently function as specialized hubs that coordinate genome replication, transcription, and particle assembly while shielding viral intermediates from host defenses. The factories of *Nucleocytoviricota*, in particular, stand out for their spatial organization, scale, and striking resemblance to cellular organelles. Their structural and biochemical complexity, along with interactions with host organelles and metabolic pathways, underscores their importance to both virology and evolutionary cell biology. As we continue to uncover the molecular underpinnings and diversity of these structures, VFs may offer profound insights into the origins of eukaryotic cellular organization and provide novel targets for broad-spectrum antiviral strategies.
